# Classification of difficult direct laryngoscopy using the VIDIAC score: a prospective external validation study

**DOI:** 10.1111/anae.70382

**Published:** 2026-10-14

**Authors:** Thorsten Dohrmann, Vera Schäfersküpper, Catharina Grün, Viktor A. Wünsch, Martin Petzoldt

**Affiliations:** ^1^ University Medical Centre Hamburg‐Eppendorf Hamburg Germany

Difficulty of direct laryngoscopy has traditionally been classified by the glottic view obtained with the naked eye during laryngoscopy, using the Cormack–Lehane classification or the percentage of glottic opening (POGO) score [1, 2]. With videolaryngoscopy, however, the laryngeal view obtained and the difficulty of tracheal tube placement became uncoupled, marking a turning point from purely view‐centric grading systems towards specifically tailored composite classification tools that integrate glottic view and performance [2, 3, 4, 5].

The videolaryngoscopic intubation and difficult airway classification (VIDIAC) score is a reliable universal classification for videolaryngoscopy [3, 4, 5, 6]. It has shown excellent diagnostic performance and calibration in validation studies across different populations, clinical contexts and blade types in children and adults [3, 5, 6], making it the most rigorously validated classification tool for videolaryngoscopy to date [4, 7]. The clinical utility of the VIDIAC score has been shown in a large prospective study; documented VIDIAC scores had substantial clinical implications for future airway management planning and decision‐making [8].

The VIDIAC score was developed for videolaryngoscopy only. Although direct laryngoscopy and videolaryngoscopy rely on similar technical skills and are subject to the same underlying anatomical–functional mechanisms, it is uncertain whether it is suitable for classifying direct laryngoscopy as well. It is further unclear which VIDIAC score values might be associated with adverse events or prompt actionable changes to the primary airway strategy. This secondary analysis of a prospective study [8] aimed to externally validate the VIDIAC score for direct laryngoscopy and to determine score thresholds prompting conversion to videolaryngoscopy.

We included 1086 anaesthesia encounters in adults who underwent head and neck surgery in whom direct Macintosh laryngoscopy was applied first‐line. Twelve patients could not be analysed due to incomplete VIDIAC recordings. The primary outcome was difficult tracheal intubation with direct laryngoscopy, defined as a difficult airway alert documented in patients' health records after the difficulty was encountered. Secondary outcomes were conversion to videolaryngoscopy; multiple tracheal intubation attempts; prolonged tracheal intubation (≥ 120 s); and airway‐related adverse events.

Airway operators assessed VIDIAC predictor variables [5] and the Cormack–Lehane classification [2]. Secondary outcomes were assessed by an independent research assistant not involved in patient care. Statistical analysis was performed using R (version 4.3.2, R Foundation for Statistical Computing, Vienna, Austria).

Difficult tracheal intubation with direct laryngoscopy was recorded in 81 (8%) patients; in 45 (4%), airway operators converted to videolaryngoscopy. The area under the receiver operating characteristic curve (AUROC) for predicting difficult tracheal intubation with direct laryngoscopy was 0.92 (95%CI 0.88–0.96) for the VIDIAC score and 0.80 (95%CI 0.76–0.84) for the Cormack–Lehane classification (p < 0.001, online Supporting Information Figure S1). The AUROC for predicting conversion to videolaryngoscopy was 0.95 (95%CI 0.91–0.99) for the VIDIAC score.

The probability of a difficult airway alert being recorded after tracheal intubation and of conversion to videolaryngoscopy, increased greatly at a VIDIAC score of 2 points and particularly when a threshold of ≥ 3 points was reached (Table 1 and online Supporting Information Figure S2). Furthermore, the risk of multiple attempts, prolonged tracheal intubation and airway‐related adverse events increased significantly (4‐fold, 8‐fold and 4‐fold, respectively) once a level of 2 VIDIAC points was reached, and especially when ≥ 3 points were reached (4‐fold, 45‐fold and 10‐fold, respectively).

**Table 1:** Distribution of VIDIAC score values and their association with primary and secondary outcomes. Values are number (proportion) or odds ratio (95%CI).

VIDIAC class	Easy*		Moderate	Hard	Severe		
VIDIAC score	‐1	0	1	2	3	4	5
Incidence	779 (72.5%)	136 (12.7%)	75 (7.0%)	51 (4.7%)	20 (1.9%)	10 (0.9%)	3 (0.3%)
Primary outcome							
Difficult tracheal intubation with direct laryngoscopy	7	5	12	25	19	10	3
	Reference		14.3 (6.1–33.5)	72.4 (33.5–165.0)	2408 (461–44,622)		
Secondary outcomes							
Conversion to videolaryngoscopy	2	1	4	13	15	8	2
	Reference		17.1 (3.7–88.4)	104.0 (32.0–468.0)	950 (272–4637)		
Multiple tracheal intubation attempts	73	24	24	16	8	3	0
	Reference		4.0 (2.3–6.7)	3.9 (2.0–7.1)	4.2 (1.9–8.8)		
Prolonged tracheal intubation; ≥ 120 s	73	28	24	25	15	10	3
	Reference		3.9 (2.2–6.5)	7.7 (4.3–13.9)	45.0 (18.4–135.0)		
Airway‐related adverse events	11	2	2	3	3	1	0
	Reference		1.9 (0.3–7.1)	4.3 (1.0–14.0)	9.6 (2.6–29.0)		

* Reference category.

Difficult tracheal intubation with direct laryngoscopy was documented most frequently if epiglottis movement remained impaired (45/81, 56%) or the vocal cords were still not visible (35/81, 43%) despite increased lifting force (Fig. 1). Conversion to videolaryngoscopy occurred predominantly in patients with multiple VIDIAC characteristics: 40/45 (89%) had at least two characteristics and 28/45 (62%) had at least three. The mechanism prompting conversion to videolaryngoscopy most frequently was that the vocal cords could not be seen due to persistently impaired epiglottis movement despite increased lifting force (21/45, 47%).

**Figure 1:**
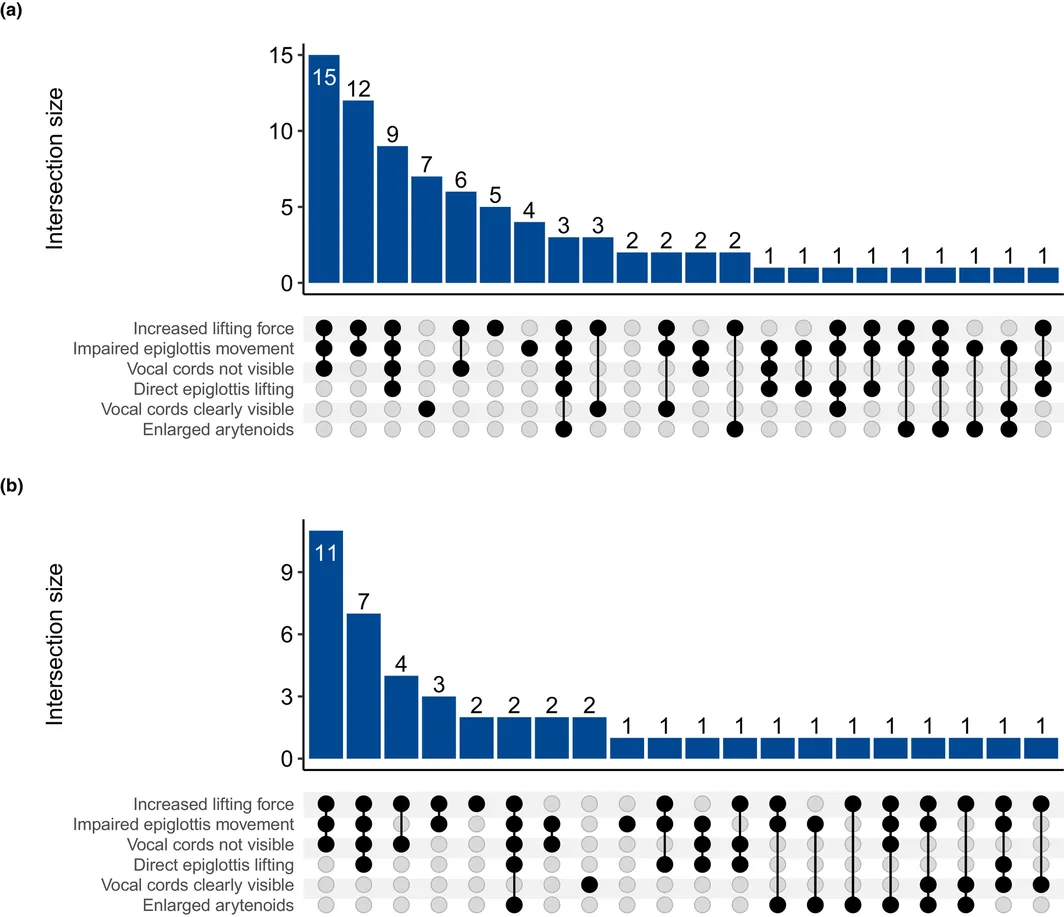
Factors contributing to the decision to (a) issue a difficult airway alert after direct laryngoscopy and (b) convert to videolaryngoscopy, illustrated on UpSet plots. The sizes of the intersections are shown as bar charts.

The VIDIAC score reliably classified direct laryngoscopy and outperformed the Cormack–Lehane classification. It is a useful tool for documenting difficult direct laryngoscopy (e.g. using yellow flags for VIDIAC score 2 and red flags for VIDIAC score ≥ 3). Moreover, it can be used in real time during airway management to support decision‐making and to adapt the airway management strategy in a timely manner. Score characteristics are strong predictors of adverse events.

Conversion to videolaryngoscopy should be considered at a VIDIAC score of 2 and particularly at scores ≥ 3. Failure to see the vocal cords due to impaired epiglottis movement despite forceful engagement of the vallecular fold remains the leading mechanism underlying difficulty with both direct and indirect Macintosh laryngoscopy [1].

In conclusion, we validated the VIDIAC score for direct laryngoscopy, which in turn can support ad hoc decision‐making during airway management. Score values of 2 and especially of ≥ 3 should prompt an adaptation of the airway management strategy to prevent multiple and prolonged tracheal intubation attempts and adverse outcomes.

## Supporting information


**Figure S1.** Receiver operating characteristic curves for the VIDIAC score and the Cormack–Lehane classification for classifying difficult tracheal intubation with direct laryngoscopy.
**Figure S2.** Distribution of the VIDIAC score values.

**Table S1.** Characteristics of the study cohort.
**Table S2.** Study outcomes.
