# Supplementary material for: Classification of difficult direct laryngoscopy using the VIDIAC score: a prospective external validation study
Source: Anaesthesia. 2026 Oct 14;81(12):1758–60. doi: 10.1111/anae.70382 (PMC13743683; doi:10.1111/anae.70382)
Supplement: Supplementary file 1 [file ANAE-81-1758-s001.docx]

**Table S1** Characteristics of the study cohort. Values are number (proportion) or median (IQR [range])

| **Characteristic** | **Overall N = 1,074** | **Not difficult*^*^* N = 993** | **Difficult*^*^* N = 81** |
| --- | --- | --- | --- |
| Sex; female | 472 (43.9%) | 445 (44.8%) | 27 (33.3%) |
| Age | 50 (33-64  [18-97]) | 49 (33-64  [18-97]) | 53 (46-64  [18-83]) |
| ASA score ≥3 | 216 (20.1%) | 190 (19.1%) | 26 (32.1%) |
| Previous difficult mask ventilation | 21 (6.3%) | 17 (5.6%) | 4 (4.9%) |
| Previous difficult tracheal intubation | 8 (2.4%) | 4 (1.3%) | 4 (4.9%) |
| Simplified Airway Risk Index ≥4 | 144 (13.4%) | 123 (12.4%) | 21 (25.9%) |
| Type of surgery |  |  |  |
| Microlaryngoscopy | 117 (10.9%) | 110 (11.1%) | 7 (8.6%) |
| Pharyngeal, laryngeal & tracheal surgery | 63 (5.9%) | 54 (5.4%) | 9 (11.1%) |
| Ear, nose or sinus surgery | 389 (36.2%) | 368 (37.1%) | 21 (25.9%) |
| Endocrine glands | 61 (5.7%) | 56 (5.6%) | 5 (6.2%) |
| Head and neck surgery | 242 (22.5%) | 225 (22.7%) | 17 (21.0%) |
| Mandibular surgery | 118 (11.0%) | 106 (10.7%) | 12 (14.8%) |
| Dentoalveolar surgery | 64 (6.0%) | 54 (5.4%) | 10 (12.3%) |
| Others | 20 (1.9%) | 20 (2.0%) | 0 |

*refers to whether the primary study outcome `difficult tracheal intubation with direct laryngoscopy´ was achieved or not.

**Table S2** Study outcomes. Values are median (IQR [range]) or number (proportion).

| **Characteristic** | **Overall N = 1,074** | **Not difficult^*^ N = 993** | **Difficult^*^ N = 81** |
| --- | --- | --- | --- |
| Glottic view [5, 6, 8] |  |  |  |
| Vocal cords completely visible (I) | 551 (51.3%) | 546 (55.0%) | 5 (6.2%) |
| Part of the cords visible (IIa) | 345 (32.1%) | 336 (33.8%) | 9 (11.1%) |
| Posterior cords only just visible (IIb) | 131 (12.2%) | 102 (10.3%) | 29 (35.8%) |
| Arytenoids but not cords visible (IIc) | 25 (2.3%) | 7 (0.7%) | 18 (22.2%) |
| Epiglottis but no glottis visible (III) | 20 (1.9%) | 2 (0.2%) | 18 (22.2%) |
| Laryngeal structures not visible (IV) | 2 (0.2%) | 0 | 2 (2.5%) |
| Multiple intubation attempts | 148 (13.8%) | 115 (11.6%) | 33 (40.7%) |
| Tracheal intubation time | 37 (21-85 [7-982]) | 33 (20-68 [7-813]) | 277 (99-374 [23-982]) |
| Airway-related adverse events | 22 (2.0%) | 12 (1.2%) | 10 (12.3%) |
| Oesophageal intubation | 5 (0.5%) | 3 (0.3%) | 2 (2.5%) |
| Airway swelling / oedema | 2 (0.2%) | 2 (0.2%) | 0 |
| Bleeding | 14 (1.3%) | 7 (0.7%) | 7 (8.6%) |
| Dental damage | 5 (0.5%) | 2 (0.2%) | 3 (3.7%) |

*refers to whether the primary study outcome `difficult tracheal intubation with direct laryngoscopy´ was achieved or not.
