# Supplementary material for: Classification of difficult direct laryngoscopy using the VIDIAC score: a prospective external validation study
Source: Anaesthesia. 2026 Oct 14;81(12):1758–60. doi: 10.1111/anae.70382 (PMC13743683; doi:10.1111/anae.70382)
Supplement: Supplementary file 2 [file ANAE-81-1758-s002.docx]

**Figure S1** Receiver operating characteristic curves for the VIDIAC score (red) and the Cormack–Lehane classification (blue) for classifying difficult tracheal intubation with direct laryngoscopy.

**Figure S2** Distribution of the VIDIAC score values: (A) incidences of VIDIAC score values during 1074 encounters with direct laryngoscopy; and probability (blue dot) with 95%CI (vertical line) of (B) difficult tracheal intubation with direct laryngoscopy and (C) conversion to videolaryngoscopy as a function of the VIDIAC score.
